# 

**Published:** 1897-04

**Authors:** 


### MEDICAL ASSOCIATION OF GEORGIA.

The Forty-Eighth Annual Session of the Association will be held in Macon, April 21, 22, and 23. Following is a partial list of papers promised:

"Meddlesome Instrumentation in Urethral Diseases," W. L. Champion, M.D., Atlanta; "Cystitis," F. M. Ridley, M.D., LaGrange; "An Act to Create a Commissioner of Health and Drainage for the State of Georgia," J. C. LeHardy, M.D., Savannah; "A Study of the Refraction of One Thousand Eyes," C. H. Peete, M.D., Macon; "Chorea," Hugh Hagan, M.D., Atlanta; "Obstetric Surgery," Jos. Eve Allen, M.D., Augusta; "Entero-Colitis of Infancy," M. A. Clarke, M.D., Barnesville; "Hernia," W. F. Westmoreland, M.D., Atlanta; "The Treatment of Pleuritis," Newt Anderson, M.D., Eudora; "Prevention of Tuberculosis," J. S. Todd, M.D., Atlanta; "Report of Case of Central Laceration of Perineum during Labor," R. P. Cortelyou, M.D., Marietta; "Fractures of the Skull, with Report of Cases," W. S. Elkin, M.D., Atlanta; "The Induction of Premature Labor in the Interest of the Mother and the Child," E. R. Corson, M.D., Savannah; "Lupus of the Nose, its Medical and Surgical Treatment," Geo. Horine, M.D., Americus; "Physical Signs in Diseases of the Lungs," Louis H. Jones, M.D., Atlanta; "Puerperal Septicemia; its Cause and Treatment," J. D. Chason, M.D., Iron City; "Laparo-Hysterotomy, Report of a Successful Case and a Plea for its More Frequent Adoption," A. Moody Burt, M.D., Sparta; "The Neuroses of Children," Katherine Collins, M.D., Atlanta; "Some Unusual Cases in Abdominal and Gynecological Work,"

K. P. Moore, M.D., Macon; "Atypical Fevers," E. H. Richardson, M.D., Atlanta; "Hemorrhagic Fever," L. P. Hudson, M.D., Cochran; "Some Wash Drawings Illustrating Case," Geo. H. Noble, M.D., Atlanta; "Some Interesting Ophthalmological Cases," J. Lawton Hiers, M.D., Savannah; "Morphine and its Effects," Addison K. Bell, M.D., Madison; "The Abortive Treatment of Acute Suppurative Inguinal Adenitis by Pressure Bandage," Henry R. Slack, M.D., LaGrange; "Dermoid Cyst of the Bladder, with Report of Case," W. A. Crow, M.D., Atlanta; "Puerperal Eclampsia, with Report of Cases," S. Rumble, M.D., Goggansville; W. S. Kendrick, M.D., Atlanta; R. M. Harbin, M.D., Rome; C. D. Hurt, M.D., Atlanta; R. R. Kime, M.D., Atlanta; "Spinal Paralysis," W. J. Cox, M.D., Barnesville; A. W. Stirling, M.D., Atlanta; "A Case of Cystocele Complicating Labor," V. D. Lochhart, M.D., Maysville; "History of Epidemics," Marcus F. Carson, M.D., Griffin; "Two Cases of Malignant Tumor Affecting the Breast, and their Treatment," M. B. Hutchins, M.D., Atlanta.

Dr. Jos. Price, of Philadelphia, will read a paper before the Association, subject not yet announced.

